# Transcriptomic characterization of postnatal muscle maturation

**DOI:** 10.1242/dmm.052098

**Published:** 2025-03-03

**Authors:** Alix Simon, Sarah Djeddi, Pauline Bournon, David Reiss, Julie Thompson, Jocelyn Laporte

**Affiliations:** ^1^ Institut de Génétique et de Biologie Moléculaire et Cellulaire (IGBMC), CNRS UMR 7104, INSERM UMRS 1258, Université de Strasbourg, 67404 Illkirch, France; ^2^ Complex Systems and Translational Bioinformatics (CSTB), ICube laboratory – CNRS, Fédération de Médecine Translationnelle de Strasbourg (FMTS), Université de Strasbourg, 67000 Strasbourg, France

**Keywords:** Skeletal muscle maturation, Alternative splicing, Differential expression, Myopathy, *Lrp4*

## Abstract

Gene differential expression and alternative splicing are mechanisms that give rise to a plethora of tissue-specific transcripts. Although these mechanisms have been studied in various tissues, their role during muscle maturation is not well understood. Because this stage of development is impaired in multiple muscular diseases, we used RNA sequencing to analyze transcriptome remodeling in skeletal muscle from late embryonic stage [embryonic day (E)18.5] to adult mice (7 weeks). Major transcriptomic changes were detected, especially in the first 2 weeks after birth, with a total of 8571 differentially expressed genes and 3096 alternatively spliced genes. Comparison of the two mechanisms showed that they regulate different biological processes essential for the structure and function of skeletal muscle. Investigation of genes mutated in muscle disorders revealed previously unknown transcripts. In particular, we validated a novel exon in *Lrp4*, a gene mutated in congenital myasthenia, in mice and humans. Overall, the characterization of the transcriptome in disease-relevant tissues revealed key pathways in the regulation of tissue maturation and function. Importantly, the exhaustive description of alternative splicing and resulting transcripts can improve genetic diagnosis of muscular diseases.

## Teaser

**Summary:** This study utilizes RNA sequencing to reveal major transcriptome remodeling through differential expression and alternative splicing during postnatal skeletal muscle maturation in mice.

## INTRODUCTION

Skeletal muscle comprises approximately half of the total weight of the human body. It has diverse functions, including in breathing, mobility and metabolic activities. Muscle development has been extensively studied and found to occur in the following steps: differentiation of progenitors into myoblasts; fusion of myoblasts into multinucleated myotubes, which maturate into myofibers; and growth of myofibers, first through fusion with myoblasts issued from satellite cells then through cell-autonomous hypertrophy (Buckingham et al., 2003; Bentzinger et al, 2012; Chal and Pourquié, 2017). In mice, the differentiation and fusion of myoblasts occur in the late embryonic stage, around embryonic day (E)10.5-E12.5; formation of myofibers occurs in the fetal stage from E14.5-E17.5 until birth; myofiber hypertrophy occurs through cell fusion from birth to weaning (∼2-3 weeks); and myofiber hypertrophy is triggered by growth factors signaling from weaning to adulthood (3-7 weeks) (Biressi et al., 2007). Prenatal and postnatal muscle development and maturation are affected in a plethora of muscle diseases, including the severe congenital myopathies and muscular dystrophies (Jungbluth et al., 2018; Mercuri et al., 2019).

Postnatal muscle development and maturation is governed by multiple mechanisms, including external cues, cellular pathways and reconfiguration of molecular composition. Among these mechanisms, transcriptome remodeling is key and includes specific regulation of gene expression and alternative splicing (AS). AS is the process by which one gene can lead to different transcripts and subsequently potential protein isoforms. In recent years, next-generation sequencing technologies have allowed the characterization of changes in gene expression and/or AS during skeletal muscle development in different experimental systems and animal models, especially concerning muscle cell differentiation (Brinegar et al., 2017; Spletter et al., 2018; Dos Santos et al., 2023; Wang et al., 2023).

Here, we report an extensive characterization of the transcript expression and splicing found in mouse skeletal muscle during postnatal maturation, focusing particularly on genes associated with myopathies. Using bulk RNA sequencing (RNA-seq), we studied key points in muscle maturation: the end of the embryonic development at E18.5 just before birth, the maturation of the muscle by fusion of satellite cells at 2 weeks, and the mature adult muscle at 7 weeks. Specific patterns of gene expression and splicing were identified. Additionally, the transcriptome characterization of myopathy-associated genes revealed distinct features and previously unknown exons associated with these genes.

## RESULTS

### Morphological hallmarks of postnatal skeletal muscle maturation

**Fig. 1.**
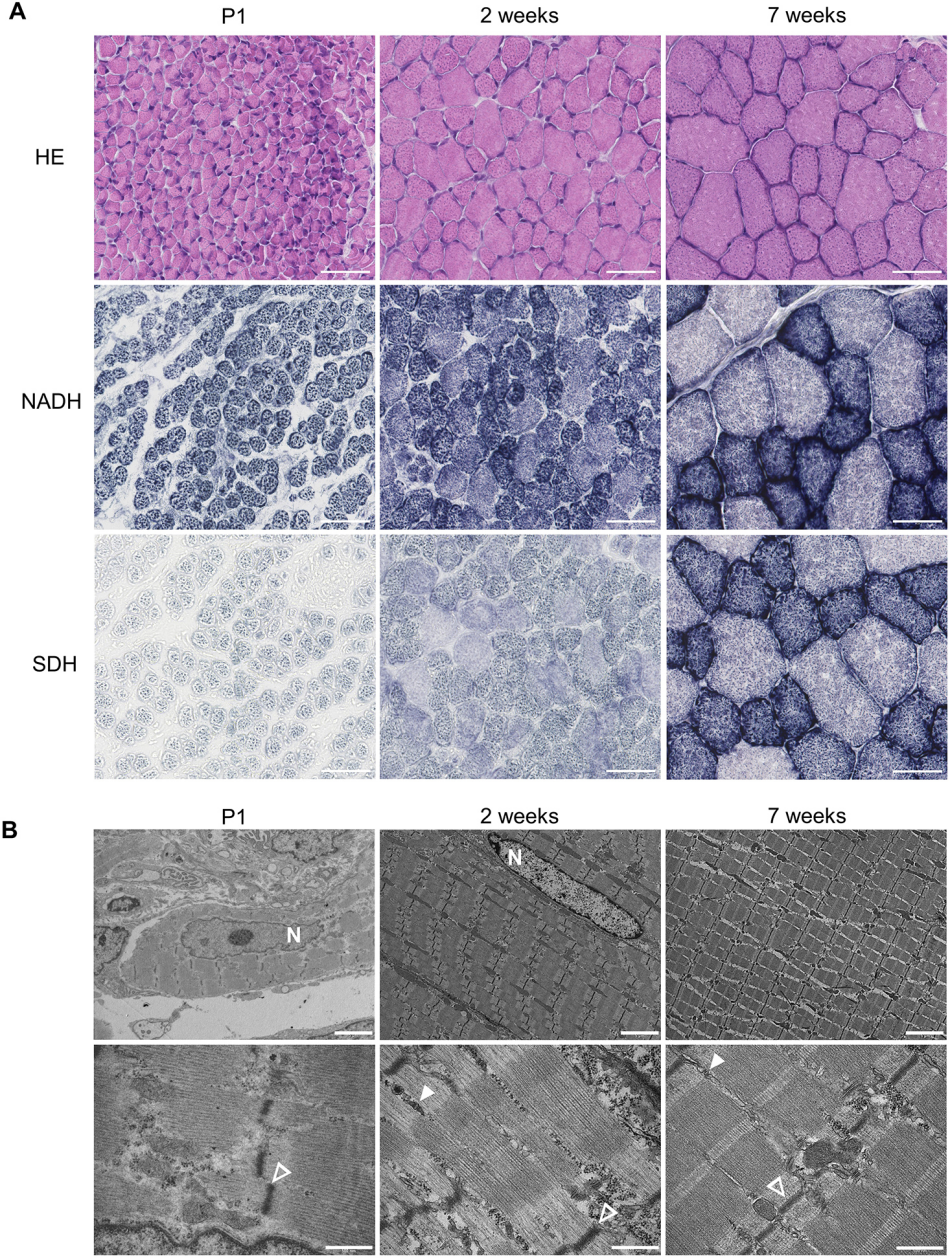
**Morphological hallmarks of postnatal skeletal maturation in mice.** (A) Representative histology of tibialis anterior muscle at postnatal day (P)1, 2 weeks and 7 weeks with Hematoxylin and Eosin (HE) staining (top row), nicotinamide adenine dinucleotide (NADH) staining (middle row) and succinate dehydrogenase (SDH) staining (bottom row). Scale bars: 50 μm. (B) Representative electron microscopy of skeletal muscle at P1, 2 weeks and 7 weeks. ‘N’ indicates nuclei, empty arrowheads indicate Z-discs, and filled arrowheads indicate triads. Scale bars: 5 μm (top row) and 500 nm (bottom row). *n*=3 for each age.

To understand the morphological changes taking place during postnatal muscle development in mice, we first performed tibialis anterior muscle histology at 1 day [postnatal day (P)1], 2 weeks and 7 weeks after birth (*n*=3 for each age). At P1, Hematoxylin and Eosin (HE) staining revealed small and rounded myofibers with occasionally internalized nuclei, which are hallmarks of immature myofibers (Fig. 1A, top row). Over time, the diameter of myofibers increased, and they achieved their characteristic polygonal shape at 2 weeks, with further development observed at 7 weeks. From 2 weeks, nuclei were positioned under the sarcolemma, as expected in mature myofibers. Oxidative staining was carried out for nicotinamide adenine dinucleotide (NADH) and succinate dehydrogenase (SDH). These stains were used to assess the internal architecture of myofibers and their metabolic activity, and to differentiate between oxidative slow fibers (dark) and glycolytic fast fibers (light). NADH staining revealed homogeneously stained myofibers at P1, whereas the two fiber types were distinguishable at 2 weeks and even more so at 7 weeks (Fig. 1A, middle row). SDH staining was used to compare the mitochondrial oxidative activity at the different ages. At P1, only weak oxidative activity was observed, and increased with time at 2 weeks and 7 weeks, also revealing the two populations of myofibers (Fig. 1A, bottom row). Finally, electron microscopy (EM) was used to investigate the ultrastructure of maturating myofibers (Fig. 1B). Z-discs, A-bands and I-bands were observed at all ages, but were better defined at 2 weeks and 7 weeks, supporting the premise that sarcomeres are already formed at birth. At P1, sarcomeres and Z-discs were misaligned, and no triads were observed. Sarcomere alignment and triads were observed from 2 weeks. Overall, these investigations highlight important changes in the intracellular structures, organelle organization and metabolic profile during postnatal muscle maturation.

### Transcriptome remodeling impacts more genes during early postnatal maturation than during late postnatal maturation

**Fig. 2.**
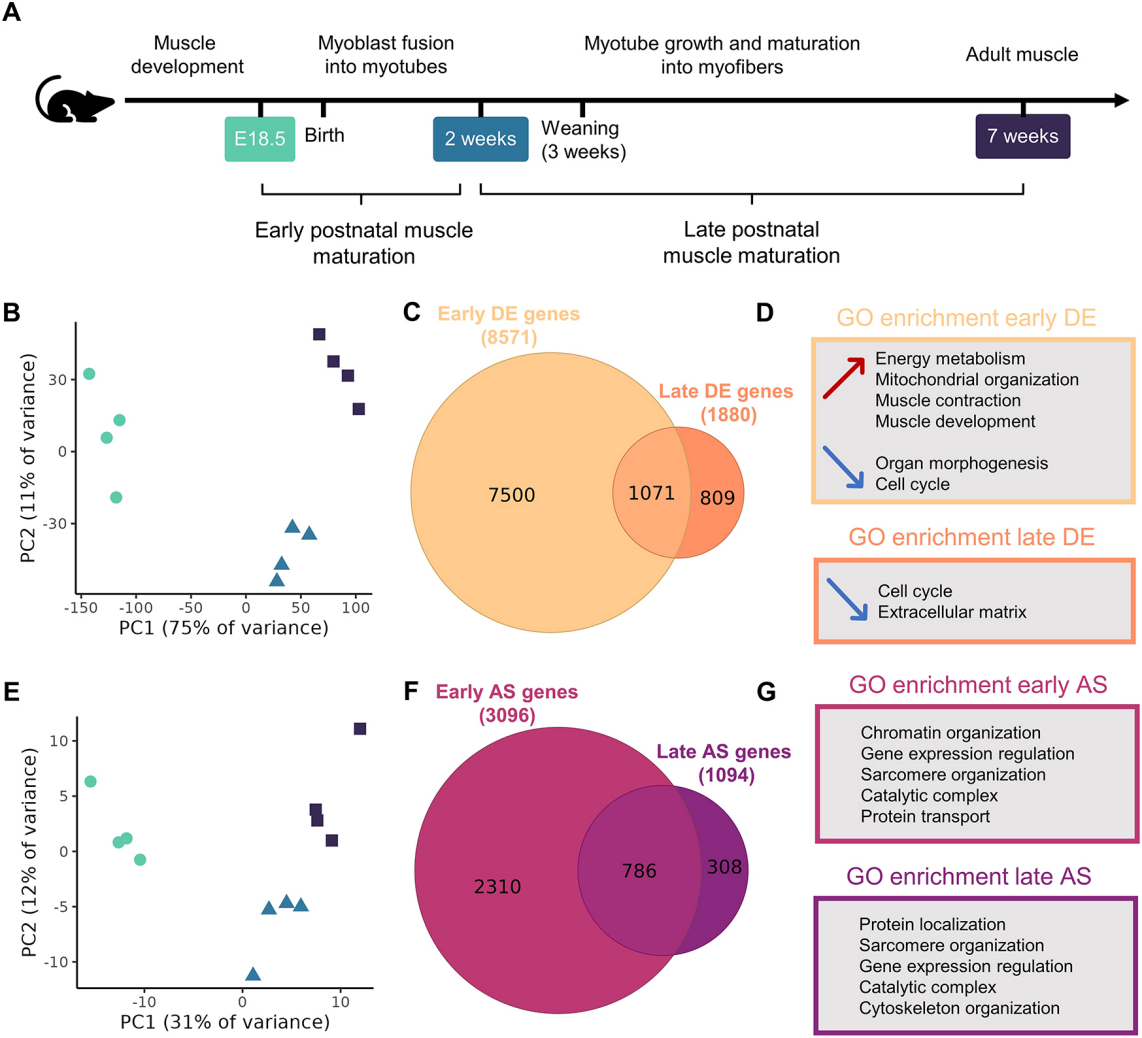
**Alternative splicing (AS) and differential expression (DE) impact more genes during early postnatal muscle maturation than during late postnatal muscle maturation.** (A) Stages of postnatal muscle maturation. (B) Principal component (PC) analysis of the samples based on gene expression. (C) Proportional Venn diagram of differentially expressed genes during early and late maturation. (D) Main enriched Gene Ontology (GO) terms in differentially expressed genes during early and late maturation. Arrows next to the enriched GO terms indicate that they are enriched in upregulated genes (red upward-pointing arrow) or downregulated genes (blue downward-pointing arrows). (E) PC analysis of the samples based on AS event percentage spliced in (PSI) values. (F) Proportional Venn diagram of alternatively spliced genes. (G) Main enriched GO terms in alternatively spliced genes during early and late maturation. Tibialis anterior muscle was used at 2 weeks and 7 weeks, and hindlimb muscles were used at embryonic day (E)18.5. *n*=4 for each age.

We hypothesized that the normal postnatal maturation of skeletal muscle is driven by transcriptome remodeling through either gene differential expression (DE) or AS. We performed 100 bp paired-end bulk RNA-seq at key timepoints during postnatal muscle maturation in male wild-type (WT) mice: at E18.5 (just before birth) in hindlimb muscles, and at 2 weeks and 7 weeks in the tibialis anterior. For each age, four biological replicates were included. Differential gene expression and splicing analyses were carried out between E18.5 and 2 weeks for early-stage postnatal maturation, and between 2 weeks and 7 weeks for late-stage postnatal maturation (Fig. 2A).

Principal component (PC) analysis based on gene expression separated the different timepoints (Fig. 2B). Indeed, PC1 and PC2 revealed three distinct groups of samples corresponding to E18.5, 2 weeks and 7 weeks. In particular, PC1 (75% of variance) separated E18.5 samples from the other two groups. In terms of DE, a total of 8571 genes were differentially expressed during the early stage between E18.5 and 2 weeks, and 1880 genes were differentially expressed during the late stage between 2 weeks and 7 weeks, with a threshold of absolute log2 fold change (log2FC) higher than 1 and an adjusted *P*-value <0.05 (Fig. 2C; Fig. S1, Tables S1 and S2). During early maturation, differentially expressed genes were evenly divided between upregulation and downregulation, whereas over 70% of late-stage differentially expressed genes were downregulated (Fig. S2A). Additionally, 57% (1071 genes) of the late-stage differentially expressed genes were also differentially expressed during early maturation (Fig. 2C). Among these genes, 77% exhibited a consistent evolution over time, whereas the rest showed opposite DE between early and late stage (Fig. S2A,B). Enrichment analysis of Gene Ontology (GO) terms revealed different processes affected at each stage (Fig. 2D; Fig. S3, Tables S3-S6). Early maturation was characterized by upregulation of genes related to muscle structure and mitochondrial organization, muscle contraction and energy metabolism, and by downregulation of genes implicated in organ morphogenesis, synapse assembly, cell cycle and cell adhesion processes. During late maturation, there was mainly downregulation of genes associated with cell cycle- and extracellular matrix-related processes.

We then investigated AS by identifying AS events with significant differential inclusion between the different timepoints. PC analysis based on percentage spliced in (PSI) values of AS events separated the different timepoints, similarly to PC analysis of gene expression (Fig. 2E). In particular, a separation of E18.5 samples from the other ages was observed in PC1 (31% of explained variance). During early postnatal maturation, 3096 genes underwent AS (with a total of 4881 significant AS events) compared to 1094 genes during late postnatal maturation (with a total of 1593 significant AS events) (Fig. 2F; Fig. S4A). In total, 50% of the late AS events were also significantly detected during early maturation (Fig. S4A). Among these events, 66% exhibited a consistent inclusion evolution over time; the rest showed opposite differential inclusion between early and late stage (Fig. S4A,B). Over 71% of genes undergoing AS during late maturation were also modulated during early maturation, and GO enrichment analysis showed that, at both stages, alternatively spliced genes were associated with similar processes, including gene expression regulation, RNA processing, protein localization and transport, catalytic complex and sarcomere organization (Fig. 2G; Fig. S5, Tables S7 and S8). To investigate the mechanisms regulating splicing, we extracted the list of genes associated with the GO term ‘RNA splicing’ (GO:0008380) and evaluated whether these genes exhibited significant DE or AS during postnatal muscle maturation (Table S9). Of 376 genes associated with RNA splicing, 69 underwent DE during early maturation (five during late maturation), and 113 showed AS during early maturation (50 during late maturation). These findings highlight that a subset of genes associated with RNA splicing undergo significant DE and AS during postnatal muscle maturation, with pronounced activity during the early maturation phase. We then used the SplicingLore database (Polvèche et al., 2023), which correlates splicing factors with alternative exons, to identify targets of the splicing-associated genes exhibiting DE and AS during early and late maturation (Table 1). Of the 3096 genes undergoing early AS, 33% were targets of early DE splicing-associated genes and 58% were targets of early AS splicing-associated genes. Similarly, splicing-associated genes undergoing late DE and AS were linked to 23% and 55%, respectively, of the 1094 late AS genes. Therefore, DE and AS of splicing-associated genes could explain the numerous AS events observed during early and late postnatal muscle maturation. Overall, the transcriptome was highly remodeled during the first 2 weeks of postnatal maturation, both in terms of expression and in splicing changes.

**Table 1.** Analysis of the impact of DE and AS on splicing-associated genes during early and late postnatal muscle maturation

	Early DE	Late DE	Early AS	Late AS
Number of affected splicing-associated genes (GO:0008380)	69	8	113	50
Number of affected splicing-associated genes found in SplicingLore	8	2	26	15
Number of targets retrieved from SplicingLore	6264	1782	26581	15992
Number of targets exhibiting AS at the corresponding stage	1004	250	1781	596

AS, alternative splicing; DE, differential expression; GO, Gene Ontology.

### Differential gene expression and alternative splicing modulate different genes and pathways

**Fig. 3.**
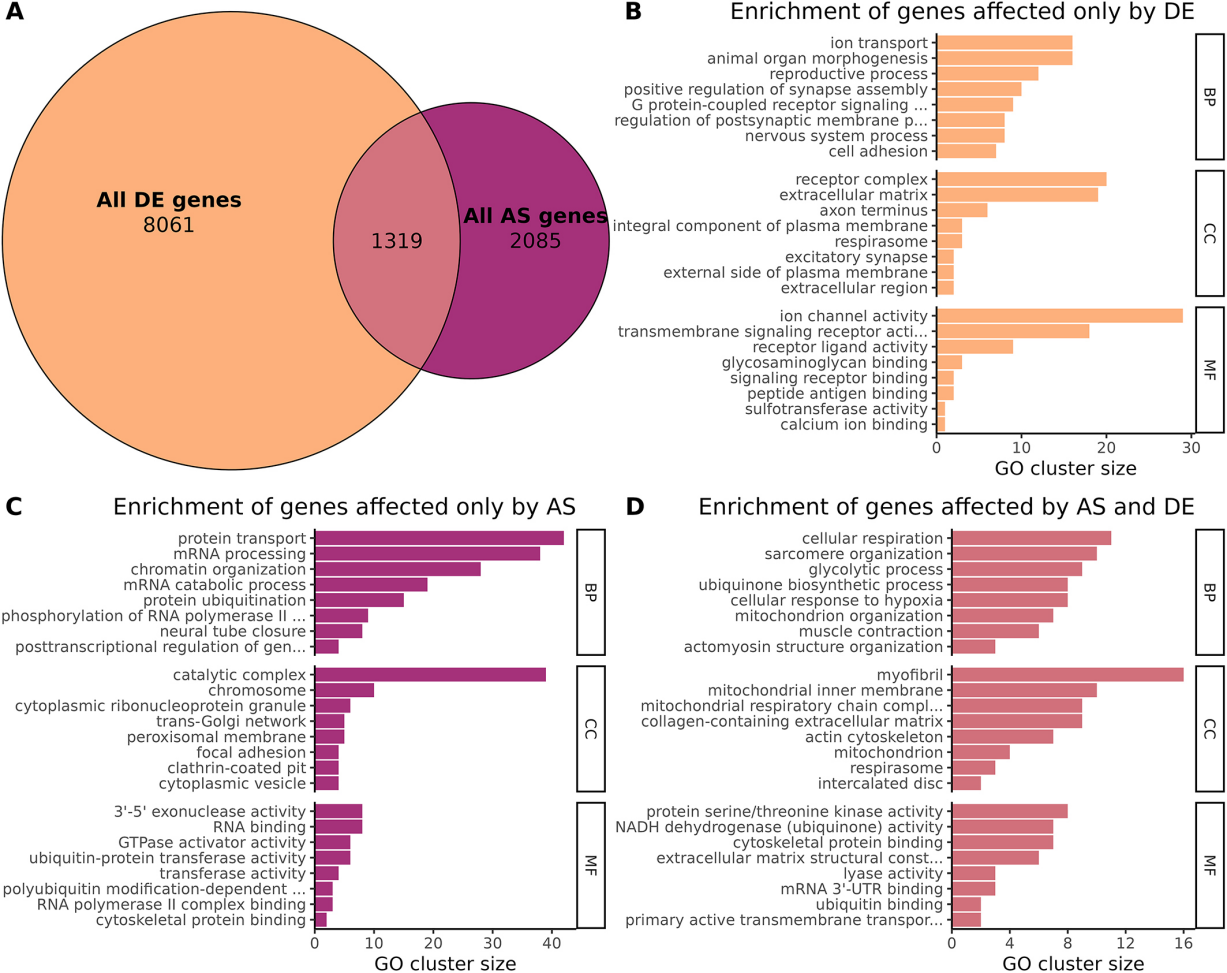
**Gene DE and AS modulate different genes and pathways in mice.** (A) Proportional Venn diagram of differentially expressed and alternatively spliced genes. (B-D) Enrichment analysis of DE-specific genes (B), AS-specific genes (C), and genes affected by both DE and AS (D). BP, biological process; CC, cellular component; MF, molecular function; UTR, untranslated region.

To investigate whether gene DE and AS regulated the same processes during postnatal maturation, we compared the list of genes that were differentially expressed or that underwent an isoform switch during early and late stages. In total, 1319 genes were both differentially expressed and alternatively spliced, which corresponded to 14% of the differentially expressed genes and 39% of the alternatively spliced genes (Fig. 3A). To identify the processes regulated by these two different mechanisms, we performed GO term enrichment analysis of genes specifically associated with DE (8061 genes) (Fig. 3B; Table S10), specific to AS (2085 genes) (Fig. 3C; Table S11) or shared by both (1319 genes) (Fig. 3D; Table S12). DE-specific genes were associated with ion transport, synapse assembly and cell adhesion, whereas AS-specific genes were associated with protein transport, mRNA processing and chromatin organization. Genes regulated by both DE and AS mechanisms were mainly linked to muscle organization and contraction, as well as to mitochondrial function. Therefore, although key muscle processes were regulated by both AS and gene DE, nervous system processes that could be linked to the establishment of the neuromuscular junction were specifically regulated by DE, whereas AS regulated transcription-related processes.

### Transcriptomic characterization of myopathy-associated genes during muscle maturation

**Fig. 4.**
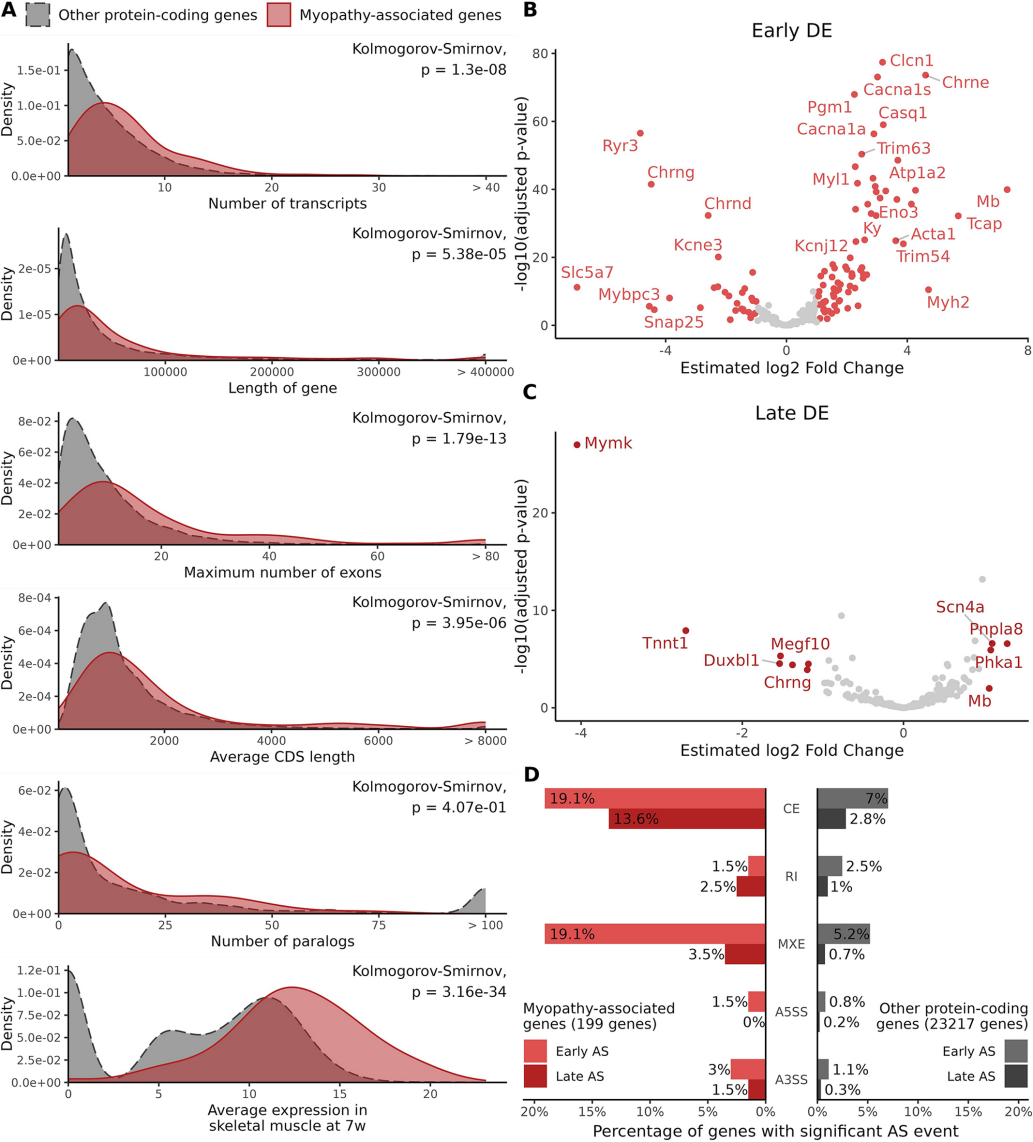
**Transcriptomic characterization of myopathy-associated genes during muscle maturation.** (A) Distribution of the number of transcripts, gene length, maximal number of exons, average coding DNA sequence (CDS) length, number of paralogs and expression in skeletal muscle for myopathy-associated genes compared to other protein-coding genes in mouse. (B,C) Volcano plot of myopathy-associated genes during early (B) and late (C) maturation in mice. Genes with an absolute log2 fold change>1 and adjusted *P*-value<0.05 are colored in red. (D) Comparison of AS events in myopathy-associated genes and other protein-coding genes in mice. A3SS, alternative 3′ splice site; A5SS, alternative 5′ splice site; CE, cassette exon; MXE, mutually exclusive exons; RI, retained intron. The percentages of genes affected by each type AS event during early and late maturation are shown.

Next, we investigated whether the genes mutated in myopathies have specific expression patterns during skeletal muscle development. A list of 199 human myopathy genes was extracted from the GeneTable of Neuromuscular Disorders (Benarroch et al., 2020). We extracted gene characteristics – such as the number of transcripts, exons and paralogs, gene length and coding DNA sequence (CDS) length – from the Ensembl database for all protein-coding genes in mouse and human. We then compared the characteristics of myopathy genes to those of the remaining protein-coding genes found in the genome. In mouse, the 199 myopathy gene orthologs were compared with the remaining 21,601 protein-coding genes found in the murine genome. Statistical comparison of the distribution of myopathy genes and all protein-coding genes revealed that myopathy genes displayed an increased number of transcripts, gene length, maximum number of exons, average CDS length and expression level in skeletal muscle (Fig. 4A). Similar conclusions were drawn from the human data, with the addition of a significantly increased number of paralogs (Fig. S6). A correlation between gene length and number of exons and transcripts is expected. Conversely, a higher number of paralogs would suggest a partial functional redundancy and possible compensation. However, paralogs of myopathy genes are often expressed in different tissues, such as *RYR1* and *CASQ1*, which are expressed in skeletal muscles, whereas *RYR2* and *CASQ2* are expressed in cardiac muscles. Similarly, *AMPH* is expressed in brain and *BIN1* in skeletal muscle, according to the Genotype-Tissue Expression (GTEx) portal (Lonsdale et al., 2013).

Based on our RNA-seq data in mice, 105 (53%) myopathy genes were differentially expressed during early maturation, compared to only 11 (6%) myopathy genes in late maturation, suggesting that myopathy genes are preferentially implicated in early muscle maturation (Fig. 4B,C). Of note, *Scn4a*, *Mymk*, *Mb*, *Megf10*, *Chrng*, *Phka1*, *Duxbl1* and *Tnnt1* appeared to be modulated during both early and late maturation. In addition, genes coding for proteins implicated in calcium handling, myofilaments and neuromuscular junction were highly upregulated during the early stage, corresponding to the final maturation of these functions, whereas *Mymk* (myomaker; mediating the fusion of myoblasts to form myocyte syncytia) was highly downregulated at the late stage, when muscle cell fusion is finished.

During development, AS plays a crucial role in the generation of protein diversity from a single gene. We investigated the repartition of the five common types of AS events: alternative 3′ and 5′ splice sites (A3SS and A5SS, respectively), mutually exclusive exons (MXEs), retained intron (RI) and cassette exons (CEs) in myopathy-associated genes and other protein-coding genes (Table 2). For myopathy-associated genes, CE and MXE events were the most prevalent, with 149 CE events and 118 MXE events during early maturation, and 80 CE events and 40 MXE events during late maturation, similarly to other protein-coding genes. In myopathy-associated genes, many significant AS events were detected in *Neb* (127 early AS events, 59 late AS events) (Table S13). This result is consistent with the size and the complexity of this gene, which encodes transcripts containing up to 165 exons. However, only 14 early AS events and three late AS events were detected in *Ttn*, which encodes transcripts containing up to 347 exons*.* To limit the influence of *Neb* on the comparison of myopathy-associated genes with other protein-coding genes, we then considered the percentage of genes affected by each type of AS event (Fig. 4D). Myopathy-associated genes were more affected by CE (19.1% of genes affected during early maturation, 13.6% during late maturation) and MXE (19.1% of genes during early maturation, 3.5% during late maturation) events compared to other protein-coding genes (percentage of genes affected by events: early CE, 7%; late CE, 2.8%; early MXE, 5.2%; late MXE, 0.7%). Similar percentages of genes affected by A5SS, A3SS and RI events were found in myopathy genes compared to other genes. AS events can potentially modulate protein function through changes in mRNA stability, protein stability and protein domain composition, suggesting that the impacted genes have different specific functions in skeletal muscle.

**Table 2.** Number of significant AS events in myopathy-associated genes and other protein-coding genes in mice

Type of AS event	Number of events in myopathy-associated genes		Number of events in other protein-coding genes	
	Early	Late	Early	Late
CE	149	80	2547	879
RI	3	6	693	270
MXE	118	40	1845	256
A5SS	3	0	204	53
A3SS	6	3	285	82

A3SS, alternative 3′ splice site; A5SS, alternative 5′ splice site; CE, cassette exon; MXE, mutually exclusive exons; RI, retained intron.

### Known and new alternative exons in myopathy genes

To further characterize AS events in myopathy genes in mice, we focused our analysis on alternative exons, using *de novo* transcript assembly. The benefit of finding new exons is highlighted by recent data (Gómez-Oca et al., 2022) and our present results concerning alternative exons specifically expressed in skeletal muscle and impacting protein functions. For example, the alternative exon 11 of *Bin1* showed increased inclusion during muscle maturation (ΔPSI=0.107 between E18.5 and 2 weeks, ΔPSI=0.02 between 2 weeks and 7 weeks) (Fig. S7). Previous studies found that it was mainly expressed in skeletal muscle and barely in any other tissues investigated (Butler et al., 1997; Lee et al., 2002; Cowling et al., 2017). It encodes an in-frame sequence of 15 basic amino acids implicated in the regulation of an open-close conformation, impacting the interaction with other proteins such as dynamin 2 (Kojima et al., 2004). The alternative exon 12b of *Dnm2*, encoding dynamin 2, showed increased inclusion during muscle maturation (ΔPSI=0.153 between E18.5 and 2 weeks, ΔPSI=0.151 between 2 weeks and 7 weeks) and is mainly found in skeletal muscle (Fig. S8) (Gómez-Oca et al., 2022). Exclusion of exon 12b has been shown to exacerbate phenotypes of X-linked centronuclear myopathy in a mouse model (Gómez-Oca et al., 2022). Both *BIN1* and *DNM2* genes are mutated in autosomal centronuclear myopathies.

**Fig. 5.**
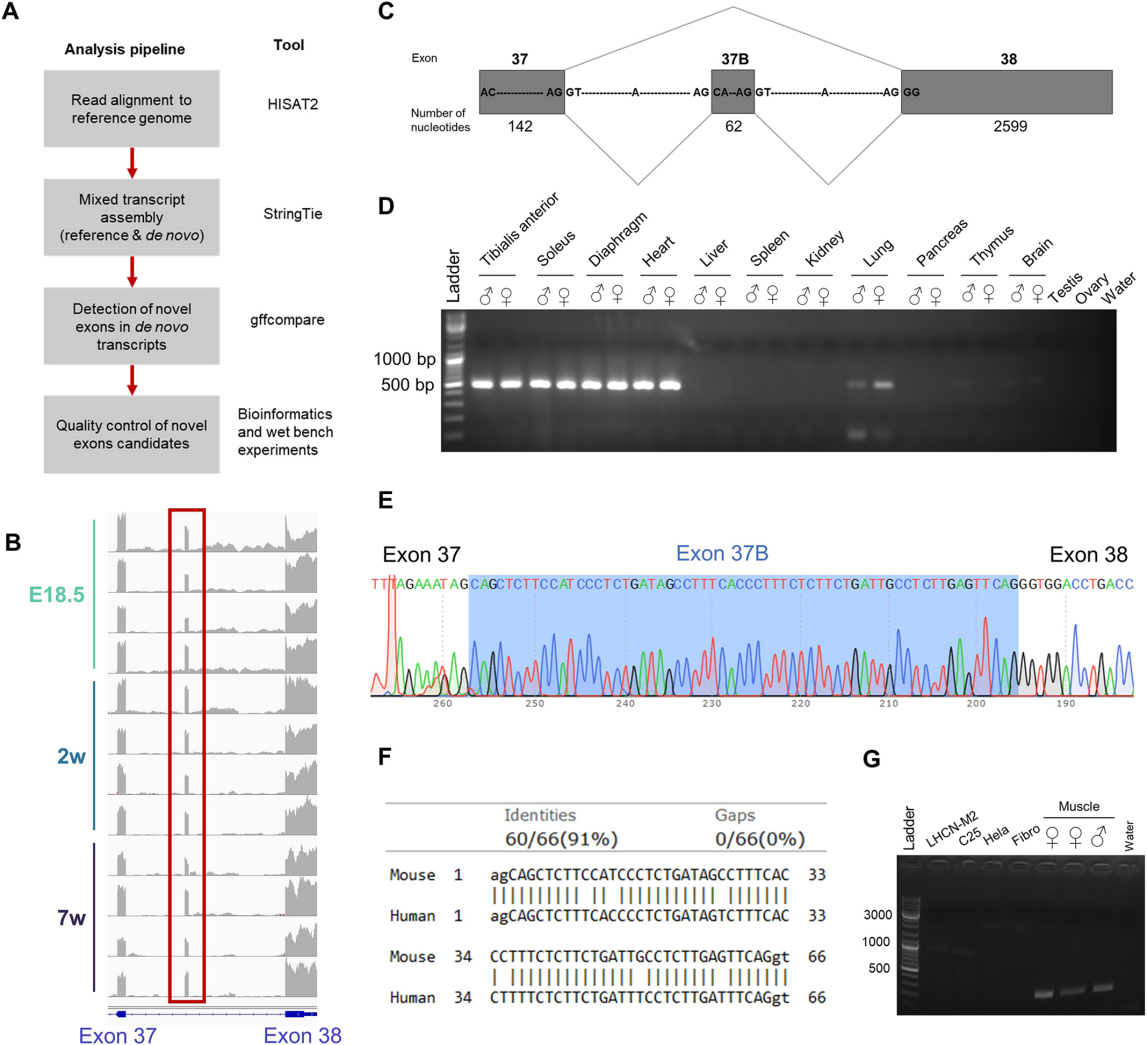
**Discovery of new exons in myopathy-associated genes.** (A) Analysis pipeline. (B) Visualization of the coverage of *Lrp4* exon 37B in the samples at E18.5, 2 weeks and 7 weeks using the Integrative Genomics Viewer (IGV). (C) Schema of exon 37B in *Lrp4*. (D) Detection of *Lrp4* exon 37b by RT-PCR gel electrophoresis in several tissues in male and female wild-type (WT) mice at 8 weeks (*n*=1 for each tissue/sex combination). (E) Sanger sequencing of the RT-PCR product. (F) Alignment of the murine 37B exon sequence with the human genome. (G) Detection of *LRP4* exon 37B by RT-PCR gel electrophoresis in myoblasts (LHCN-M2, C25), HeLa cells, fibroblasts and healthy human paravertebral muscle samples (*n*=3; two females, one male).

In addition to already known alternative exons, we also searched for previously unknown exons in myopathy genes. By performing mixed transcript assembly and comparing the *de novo* transcripts to the reference genome, we identified a total of 280 exons in myopathy-associated genes with at least one new boundary (Fig. 5A). New boundaries refer to previously unreported exon-intron junctions, altering the boundaries of existing exons or introducing new exons entirely. We then selected novel exon candidates with an average coverage greater than five reads, and that were expressed at least ten times more than the surrounding intronic background. Finally, we removed candidates without canonical splice sites, and obtained 28 potential novel exons. Each candidate was then assessed manually to compare its expression to that of the flanking exons (Fig. 5B). For example, a previously unknown *Lrp4* exon was confirmed through reverse transcription PCR (RT-PCR) and Sanger sequencing. *Lrp4* encodes a receptor for agrin and is critical for the formation and maintenance of the neuromuscular junction (Ohkawara et al., 2014). This exon 37B is located between exons 37 and 38 of *Lrp4*, and its expression level is similar to that of the flanking exons (Fig. 5B). It is expressed at all ages in this study. This exon 37B is composed of 62 nucleotides framed by canonical splice sites and introduces an early stop codon after 14 amino acids, leading to the loss of charged residues compared to the canonical protein (Fig. 5C; Fig. S9). Using RT-PCR and Sanger sequencing, we confirmed the presence of exon 37B in male and female mice at 8 weeks of age (Fig. 5D,E). This exon was highly expressed in skeletal muscles (tibialis anterior, diaphragm, soleus) and in the heart; lower expression was also detected in lung, and a faint signal was present in thymus and brain; and no expression was detected in liver, spleen, kidney, pancreas, testis and ovary (Fig. 5D). These results indicate that exon 37B is preferentially expressed in muscle. Alignment of the exon 37B sequence to the human genome revealed a match located between exons 37 and 38 of the human *LRP4* gene (Fig. 5F). The human and murine sequences showed 91% pairwise identity, with six mismatches over 66 nucleotides, and were flanked by canonical splice sites. This exon is also present in a transcript of *LRP4* predicted by automated computational analysis (XM_017017734.2) (O'Leary et al., 2016) but has never been confirmed. Using RT-PCR, we confirmed the presence of exon 37B in male and female human muscle samples, but not in LHCNM2 and C25 myoblasts, HeLa cells or fibroblasts (Fig. 5G). In conclusion, we were able to validate a new exon in *Lrp4* in mouse that is highly expressed in muscle tissues, and confirm its presence in human muscle samples. Although this exon had been computationally predicted in human, it had never been confirmed, which could be due to its muscle-specific expression pattern.

## DISCUSSION

Muscle maturation is defective in a plethora of muscle diseases and tightly regulated via transcriptome modulation (Bismuth and Relaix, 2010). In this work, we investigated the mouse transcriptome at three important key points of skeletal muscle maturation, with a particular focus on myopathy-associated genes. We showed that the transcriptome is greatly remodeled during the early stage of development, either by transcription regulation or by AS. We then detailed the differences between myopathy-associated genes and other protein-coding genes. Finally, we were able to detect and validate a novel exon in *Lrp4*, ortholog of a gene implicated in congenital myasthenic syndrome in human (Ohkawara et al., 2014).

### Transcriptome remodeling during postnatal muscle maturation

To better understand the regulation of postnatal muscle maturation, we compared histological and structural changes with DE or AS of genes. Myofiber intracellular organization is achieved within the first 2 weeks of age, as shown by the histological and electron microscopy analyses. Concomitantly, we found extensive remodeling of the transcriptome during the first 2 weeks of postnatal muscle maturation in mice compared to that during later stages. Our data are consistent with the findings of a previous study in mice (Brinegar et al., 2017), as, in both cases, DE and AS analyses revealed that the transcriptome is highly remodeled during the first 2 weeks after birth. Additionally, our study provides enhanced robustness to the findings by including biological replicates, which were not incorporated in the prior work. These observations reflect the changes averaged over many cell types, because the bulk RNA-seq on tibialis anterior (2 weeks and 7 weeks) and hindlimb muscles (E18.5) performed here did not allow observations at single-cell resolution.

Notably, we observed only a partial overlap between genes that are differentially expressed and those that are alternatively spliced. These results suggest that DE and AS control different mechanisms across time. Genes regulated by DE or by AS code for different pathways during early maturation. DE mainly impacts sarcomere and mitochondrial organization, muscle contraction and energy metabolism, and cell division, whereas AS modulates mRNA processing, protein localization, muscle development and organelle organization (Fig. 2). These findings correlate with the known maturation events, i.e. decreased cell division and increased structural organization of the myofibers, with improved muscle contraction relying on an established metabolism. Indeed, our transcriptome analysis correlates with the histological and ultrastructural investigations. The mitochondrial oxidative activity highlighted by SDH staining progressively increases with age and reaches the adult status only after weaning. This staining also highlights that mitochondrial organization matures at the early stage, in parallel with the nuclei position from internalized to subsarcolemmal depicted by HE staining and EM (Fig. 1). EM investigations also reveal that sarcomeres are fully matured by 2 weeks, with aligned Z-discs and organized triads, correlating with enrichment of sarcomere-related pathways (modulated by DE and AS) and ion channel pathways (dependent mainly on DE), respectively (Fig. 3). Indeed, efficient muscle contraction relies on these pathways.

Overall, our results indicate that DE and AS are complementary mechanisms extensively remodeling the transcriptome during postnatal muscle maturation, leading to the final myofiber organization and efficient contraction.

### Differential expression and alternative splicing in myopathy-associated genes

As discussed above, the muscle transcriptome is extensively remodeled in the first 2 weeks of postnatal maturation. Defects in perinatal muscle maturation cause a plethora of muscle diseases, especially congenital myopathies and muscular dystrophies. Interestingly, myopathy genes are generally longer than other genes, e.g. *TTN* has the longest coding sequence, encoding titin, the longest protein in mammals, and *DMD* spans the longest genomic sequence, 2.4 Mb. This suggests that myopathy genes are more susceptible to AS than other protein-coding genes, and we indeed found that they generate a higher number of different transcripts (Fig. 4).

To our knowledge, this work represents the first detailed characterization of the AS events associated with a specific disease class in normal conditions during development. The five main AS forms – including A3SS, A5SS, MXE, CE and RI – have been studied here. Although AS events vary during maturation, when considering all the genes, CE was the most predominant event, in agreement with other studies (Sultan et al., 2008; Brinegar et al., 2017). Unexpectedly, we found that myopathy genes displayed enrichment for CE and MXE events. Most myopathy genes are highly expressed in muscle, and an association between CE and MXE events and muscle has previously been reported (Hatje et al., 2017). Moreover, in the same study, a high proportion of mutations was found in these exons, which are also more specifically expressed in muscle (Hatje et al., 2017).

Overall, genes, CEs and MXEs that are expressed mainly in skeletal muscle and highly modulated during maturation appear to be excellent candidates to elucidate the missing heritability in myopathies, as nearly half of patients are still lacking genetic diagnosis. Identifying pathogenic mutations from genome or exome sequencing is challenging, and transcript usage might be tissue and age specific, meaning that clinicians could overlook relevant transcripts or exons when searching for mutations.

In this regard, the identification of previously unknown alternative exons in myopathy genes is essential. In this study, we identified and validated a novel exon in *Lrp4*. The candidate exon 37B identified in mouse was confirmed in human muscle samples. The resulting novel transcript lacks the sequence encoding the C-terminal charged region, which could affect the structure, stability and/or function of the protein.

In conclusion, the characterization of the transcriptome in disease-relevant tissues reveals novel pathways essential for the regulation of tissue maturation and function, and the exhaustive description of AS events and resulting transcripts could improve the molecular diagnosis of genetic diseases.

## MATERIALS AND METHODS

### Animal and sample collection

In this study, we analyzed WT male mice at different ages (E18.5, 2 and 7 weeks) on a 129Pas genetic background. The mice were bred in the Institut de Génétique et de Biologie Moléculaire et Cellulaire (IGBMC) animal facility in France, according to French and European regulations and approved by the institutional ethics committee (#2019101416421345). Four biological replicates were included for each age. Tibialis anterior muscles were dissected at 2 and 7 weeks, and hindlimb leg muscles were dissected at E18.5.

### RNA extraction, library preparation and sequencing

RNA was extracted from muscles with Trizol reagent (Molecular Research Center). RNA-seq libraries were prepared with a TruSeq stranded mRNA sample preparation kit (Illumina) by poly(A) selection and then sequenced on a HiSeq 4000 (Illumina) as paired-end 100 bp reads.

After sequencing, reads were preprocessed using cutadapt (version 1.10) (Martin, 2011) to remove adaptor, poly(A) and low-quality sequences (Phred quality score below 20). Reads shorter than 40 bases were excluded for downstream analysis. Then, reads were mapped to the mm39 assembly of the *Mus musculus* genome with STAR (versions 2.5.3) (Dobin et al., 2013). Quantification of gene expression was achieved using the Python package htseq-count, in the union mode with Ensembl release 104 annotations.

### Transcriptome analysis

The full code and package versions used to produce the results are provided in a Quarto notebook with associated R and bash scripts at Zenodo: https://doi.org/10.5281/zenodo.14188535.

To analyze gene DE, we processed the count table in the open-source RStudio environment for R (version 4.1.1) with Bioconductor packages. DESeq2 was used to normalize, fit and compare the data between groups (Love et al, 2014). The cutoff value for DE was set to an adjusted *P*-value <0.05 and absolute value of log2FC>1.

AS events were identified and quantified with rMATS-turbo (Shen et al., 2014; Wang et al., 2024). Statistically significant events were determined according to the recommended criteria of rMATS-turbo (Wang et al., 2024). In particular, AS events were defined as true events if the difference between two conditions was significant with adjusted *P*-value (false discovery rate) <0.01 and |ΔPSI>0.05. Raw rMATS-turbo outputs are provided at Zenodo: https://doi.org/10.5281/zenodo.14188535.

GO term enrichment analysis was performed with ClusterProfiler (Wu et al., 2021), and enriched GO terms were clustered based on their semantic similarity (Yu, 2020).

To identify new exons, we performed mixed transcript assembly with StringTie (Pertea et al., 2015), and novel exon candidates were identified with gffcompare (Pertea and Pertea, 2020). Genomics data visualization was carried out with the Integrative Genomics Viewer (IGV).

### Myopathy-associated genes

Myopathy-associated genes were retrieved from the GeneTable of Neuromuscular Disorders (Benarroch et al., 2020). The following muscle disease classes were considered for the analysis: metabolic myopathies, congenital muscular dystrophies, congenital myopathies, distal myopathies, other myopathies, congenital myasthenic syndromes, muscular dystrophies and ion channel muscle diseases. Mouse orthologs of genes causing muscle diseases, as well as characteristics of human and mouse genes (transcript information, gene length, number of paralogs) were retrieved with Biomart from Ensembl (Kinsella et al., 2011). Human gene expression level in skeletal muscle was retrieved from the GTEx portal (Lonsdale et al., 2013).

### Histology analysis

Samples were frozen in liquid nitrogen-cooled isopentane and stored at −80°C. Transversal cryosections (8 μm) were prepared and stained, and were observed using a Hamamatsu 322 NanoZoomer 2HT slide-scanner.

### EM

Samples were fixed by immersion in 2.5% glutaraldehyde and 2.5% paraformaldehyde in cacodylate buffer (0.1 M, pH 7.4), and washed in cacodylate buffer for a further 30 min. The samples were postfixed in 1% osmium tetroxide in 0.1 M cacodylate buffer for 1 h at 4°C and dehydrated through graded alcohol (50, 70, 90 and 100%) and propylene oxide for 30 min each. Samples were oriented and embedded in Epon 812 (Sigma-Aldrich). Semithin sections were cut at 2 µm, and ultrathin sections were cut at 70 nm (Leica Ultracut UCT), contrasted with uranyl acetate and lead citrate, and examined at 70 kV with a Morgagni 268D electron microscope (FEI Electron Optics) equipped with a Mega View III camera (Soft Imaging System) or with a Philips CM12 electron microscope equipped with a Gatan OneView Camera.

### RT-PCR

To validate the results obtained from the RNA-seq, RT-PCR was performed with the primers indicated in Table S14. cDNA was synthesized with Superscript IV transcriptase (Thermo Fisher Scientific).

### Cell lines and human samples used for RT-PCR

Myoblasts (LHCN-M2, C25), HeLa cells and fibroblasts were obtained from the PluriCell East platform and were properly assessed for contamination. Human paravertebral muscle tissue was donated with informed consent by three patients (two females, one male) who were undergoing surgical treatment for scoliosis. Clinical investigation was conducted according to the principles expressed in the Declaration of Helsinki.

### Protein structure visualization

Protein structure visualization was rendered using PyMOL Molecular Graphics System, Version 3.1 (Schrödinger). The protein structure of LRP4 was downloaded from the AlphaFold Protein Structure Database (accession: AF-Q8VI56-F1) (Jumper et al., 2021; Varadi et al., 2022).

## Supplementary Material

10.1242/dmm.052098_sup1 Supplementary information

Table S1. Differential expression analysis of early muscle maturation

Table S2. Differential expression analysis of late muscle maturation

Table S3. Enrichment analysis of upregulated genes during early muscle maturation

Table S4. Enrichment analysis of downregulated genes during early muscle maturation

Table S5. Enrichment analysis of upregulated genes during late muscle maturation

Table S6. Enrichment analysis of downregulated genes during late muscle maturation

Table S7. Enrichment analysis of alternatively spliced genes during early muscle maturation

Table S8. Enrichment analysis of alternatively spliced genes during late muscle maturation

Table S9. Evaluation of DE and AS in genes associated to the RNA splicing GO term

Table S10. Enrichment analysis of DE-specific genes

Table S11. Enrichment analysis of AS-specific genes

Table S12. Enrichment analysis of genes affected by DE and AS

Table S13. Number of AS events detected in myopathy-associated genes during early and late skeletal muscle maturation
