# Supplementary material for: Transcriptomic characterization of postnatal muscle maturation
Source: Dis Model Mech. 2025 Mar 3;18(2):DMM052098. doi: 10.1242/dmm.052098 (PMC11911633; doi:10.1242/dmm.052098)
Supplement: Supplementary information [file dmm-18-052098-s1.pdf]

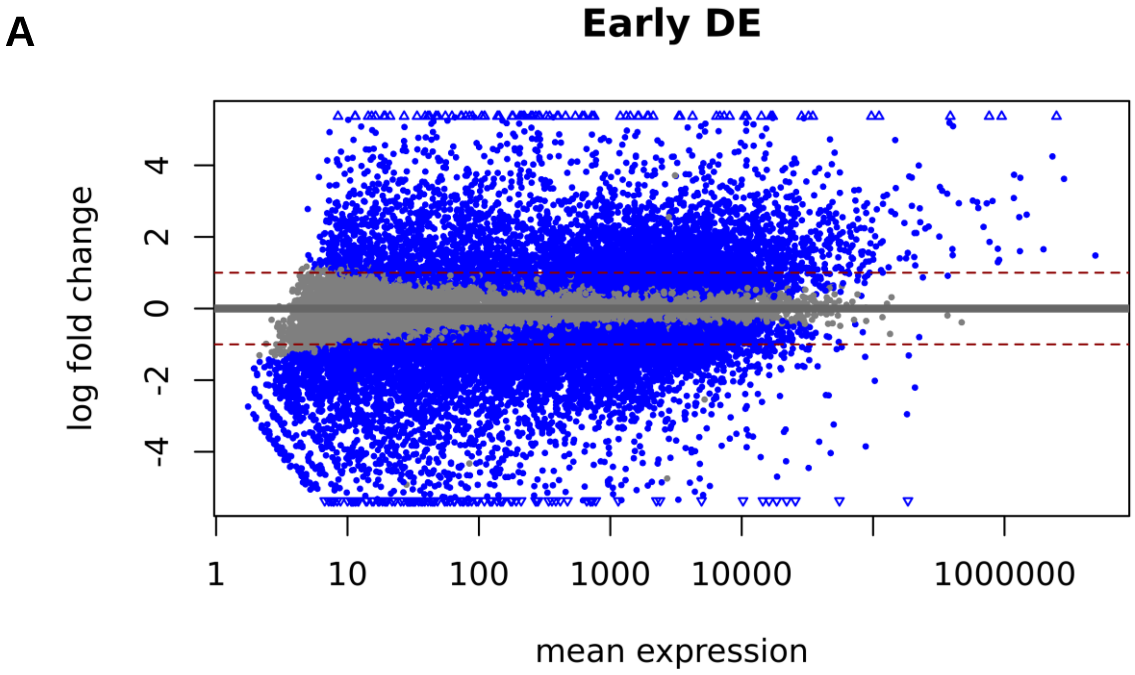

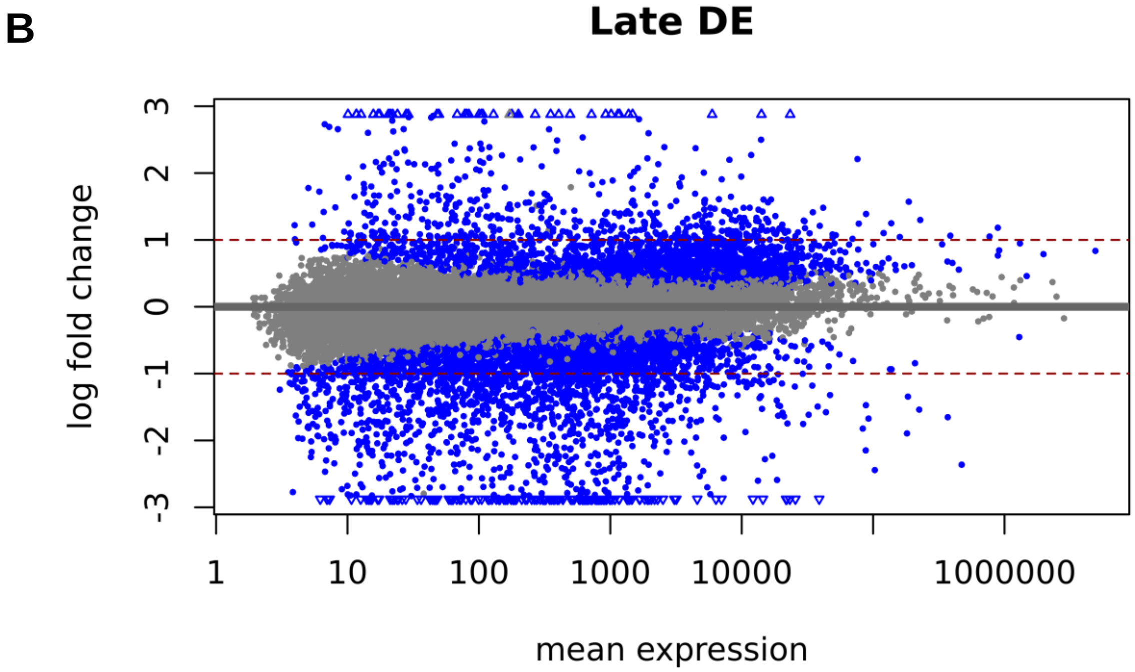

Fig. S1. MA scatter plots showing log2FC and mean normalized transcript expression.

Differential expression results are shown during **(A)** early muscle maturation, **(B)** late muscle maturation. Genes with an adjusted P-value < 0.05 are shown in blue, red dashed lines indicate |log2FC| = 1, triangles indicate genes with log2FC values exceeding the plotting limits (99th percentile of values)

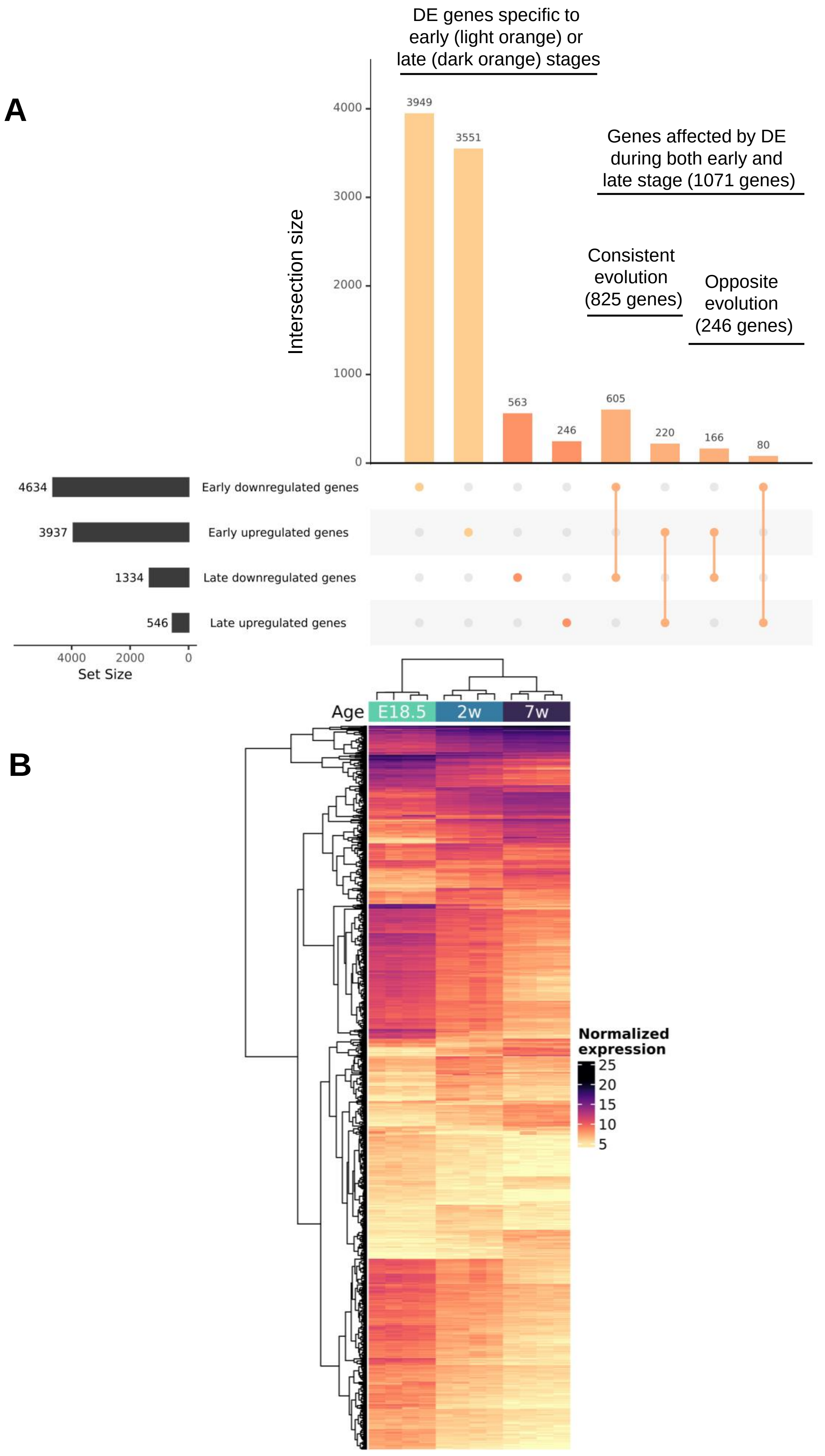

Disease Models & Mechanisms • Supplementary information

Fig. S2. Comparison of differential gene expression between early and late maturation.

(A) UpSet plot showing the intersections between genes that are up- or down-regulated during early and late muscle maturation. Dots indicate that the gene is present in the corresponding set on the left side of the bottom matrix. **(B)** Heatmap of normalized expression levels for the 1071 genes that are differentially expressed during both early and late muscle maturation

Disease Models & Mechanisms • Supplementary information

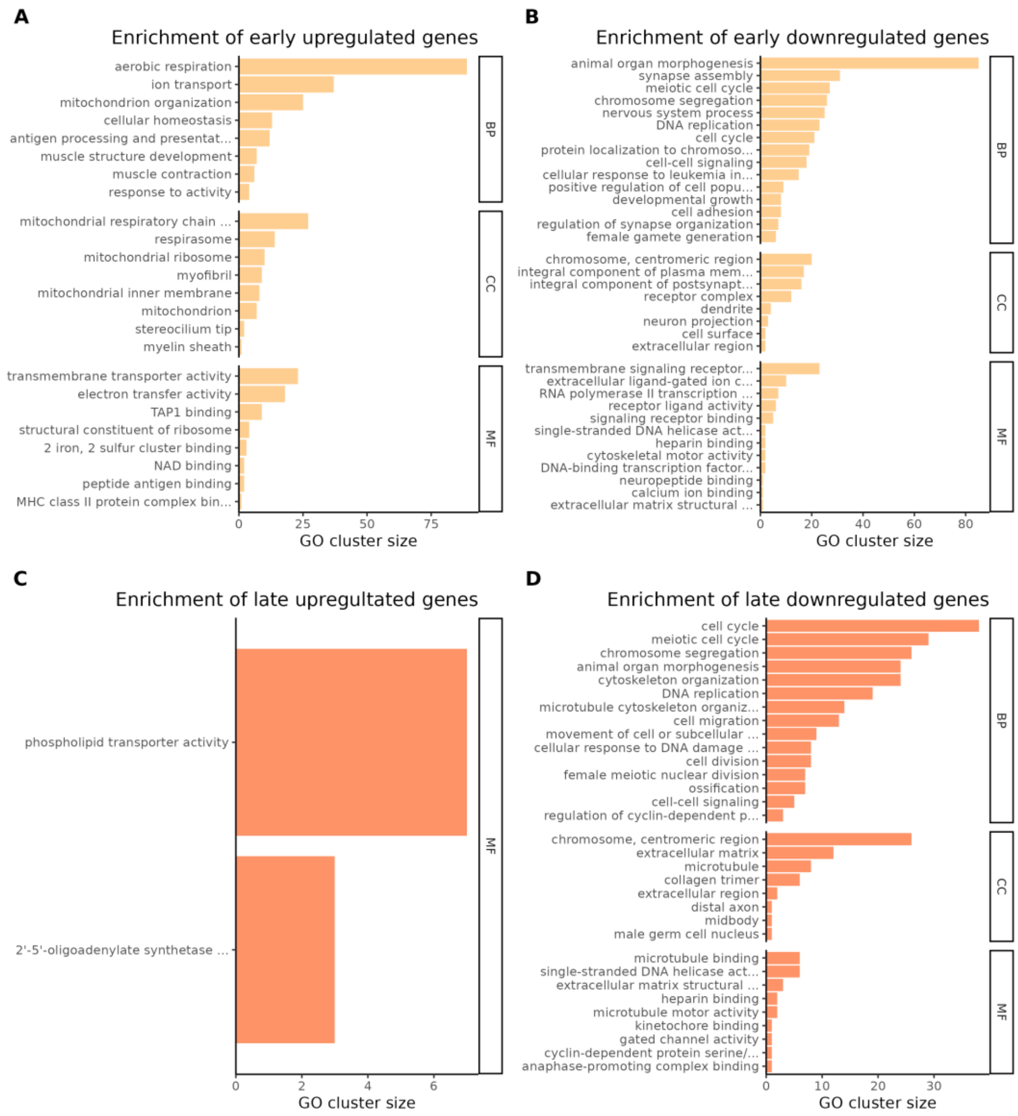

Fig. S3. Enrichment analysis of differentially expressed genes.

Analysis of genes **(A)** upregulated during early muscle maturation, **(B)** downregulated during early muscle maturation, **(C)** upregulated during late muscle maturation, **(D)** downregulated during late muscle maturation

Disease Models & Mechanisms • Supplementary information

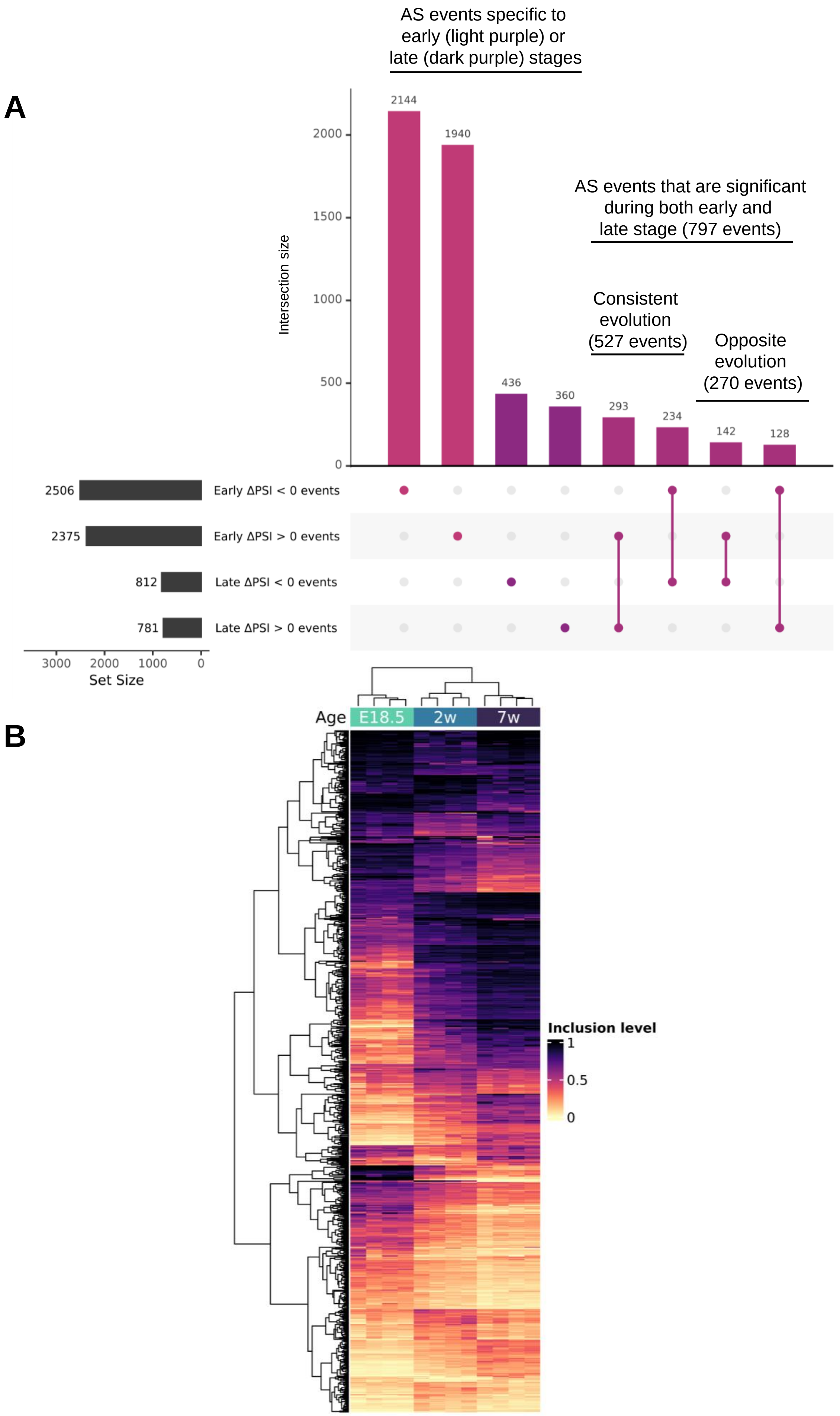

Disease Models & Mechanisms • Supplementary information

Fig. S4. Comparison of alternative splicing events between early and late maturation.

(A) UpSet plot showing the intersections between significant AS events during early and late muscle maturation. Dots indicate that the event is present in the corresponding set on the left side of the bottom matrix. **(B)** Heatmap of the 797 AS events that are significant during both early and late muscle maturation. PSI: percent spliced in, which indicates inclusion level.

Disease Models & Mechanisms • Supplementary information

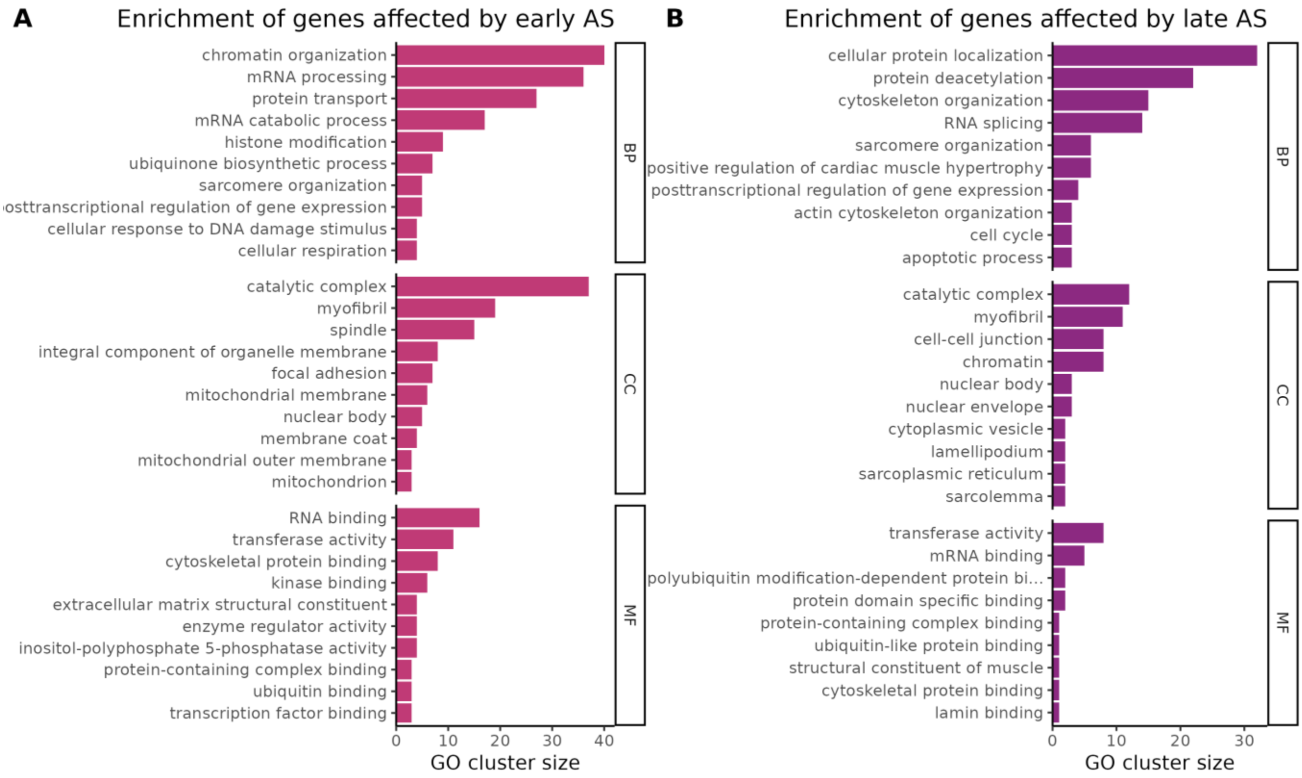

Fig. S5. Enrichment analysis of alternatively spliced genes.

Analysis was performed on genes alternatively spliced **(A)** during early muscle maturation, **(B)** during late muscle maturation

Disease Models & Mechanisms • Supplementary information

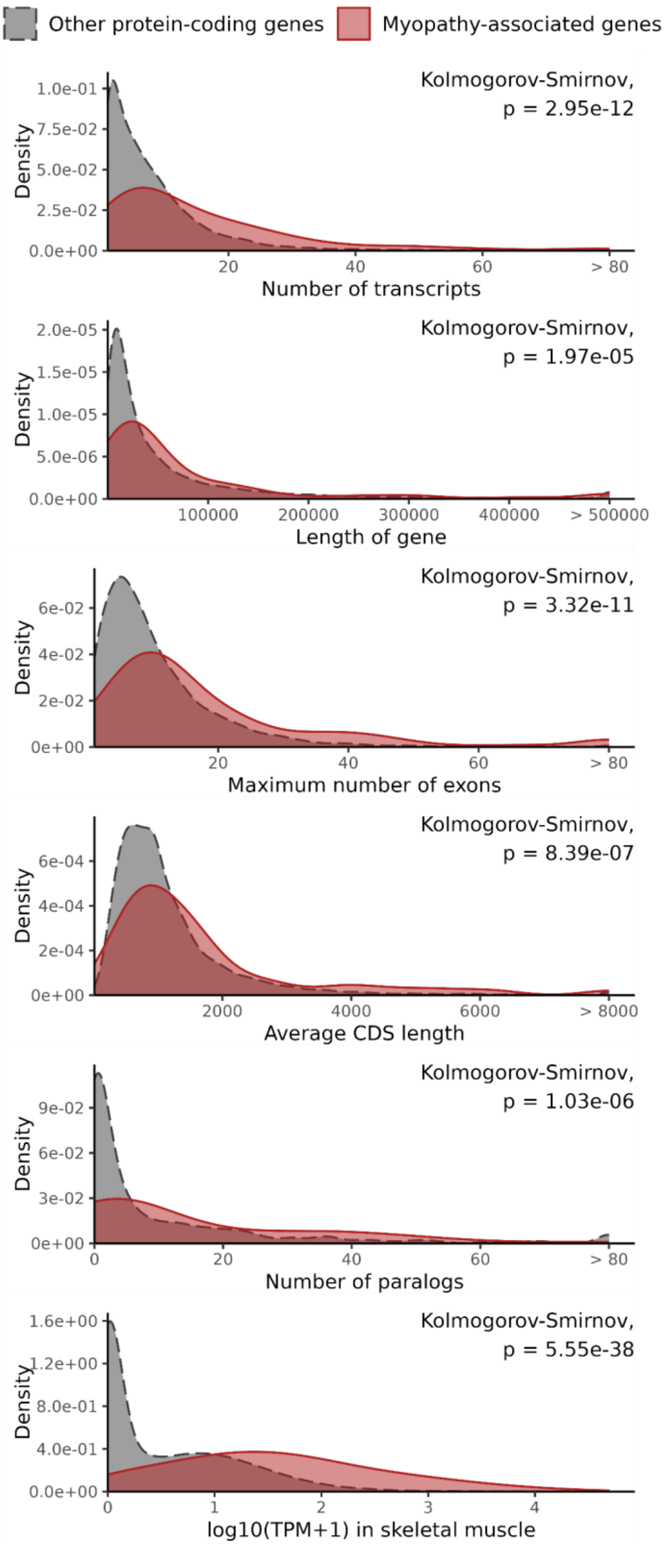

Fig. S6. Characterization of human myopathy-associated genes. Distribution of the number of transcripts, gene length, maximal number of exons, average CDS length, number of paralogs and expression in skeletal muscle in myopathy-associated genes compared to other protein-coding genes in human.

Disease Models & Mechanisms • Supplementary information

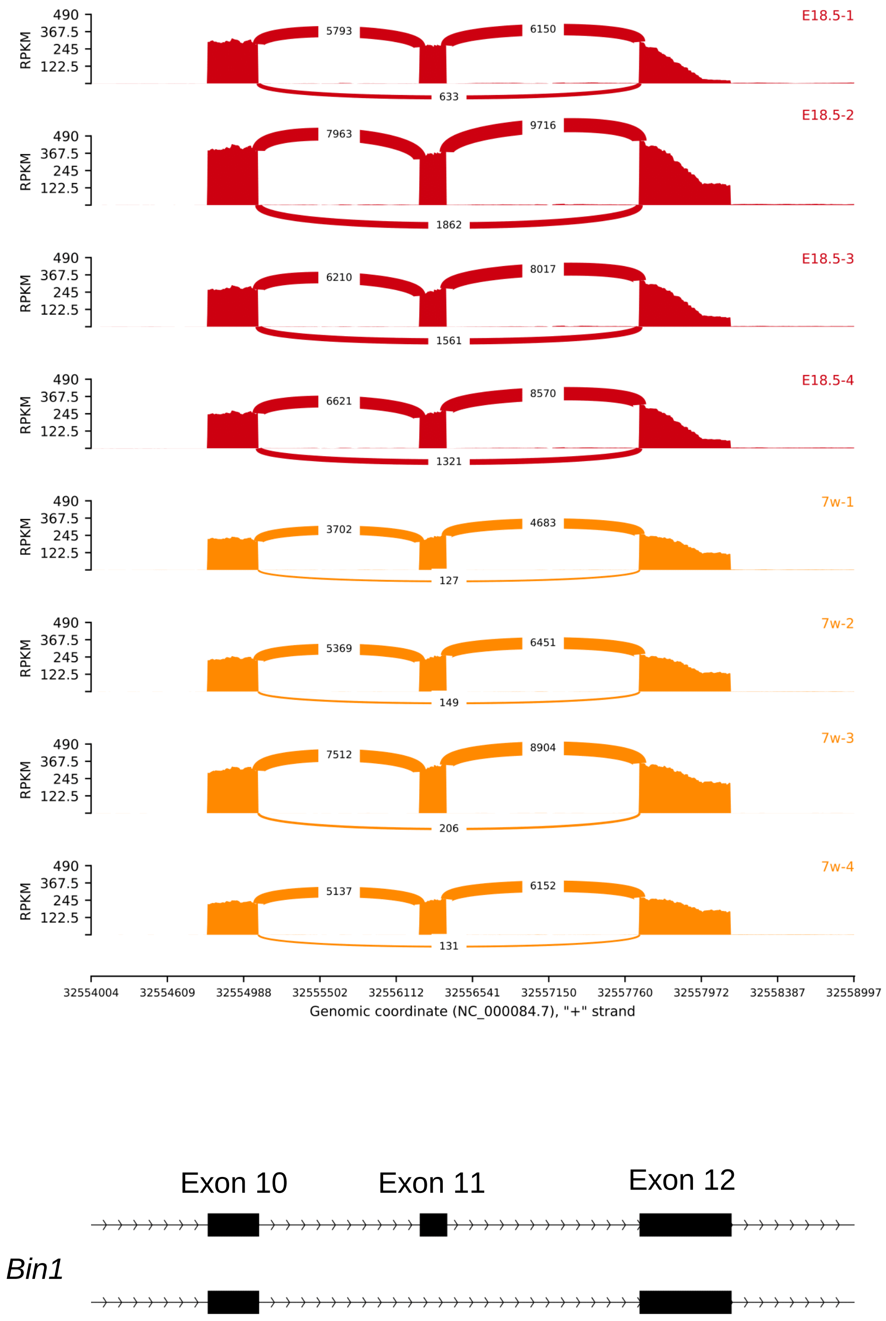

Fig. S7. Sashimi plots showing increased inclusion of exon 11 in *Bin1* between E18.5 and 7w.

Disease Models & Mechanisms • Supplementary information

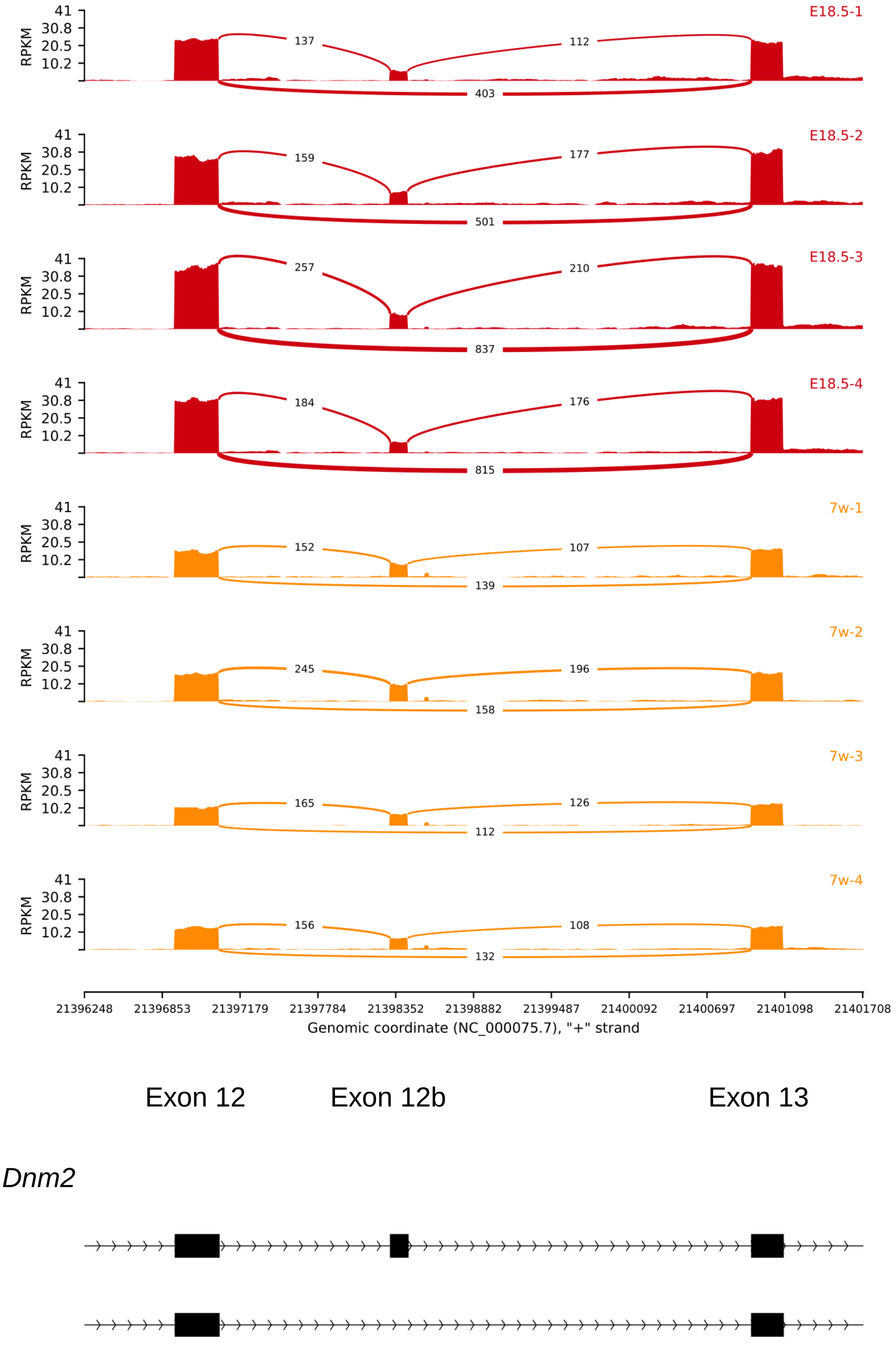

Fig. S8. Sashimi plots showing increased inclusion of exon 12b in *Dnm2* between E18.5 and 7w.

Disease Models & Mechanisms • Supplementary information

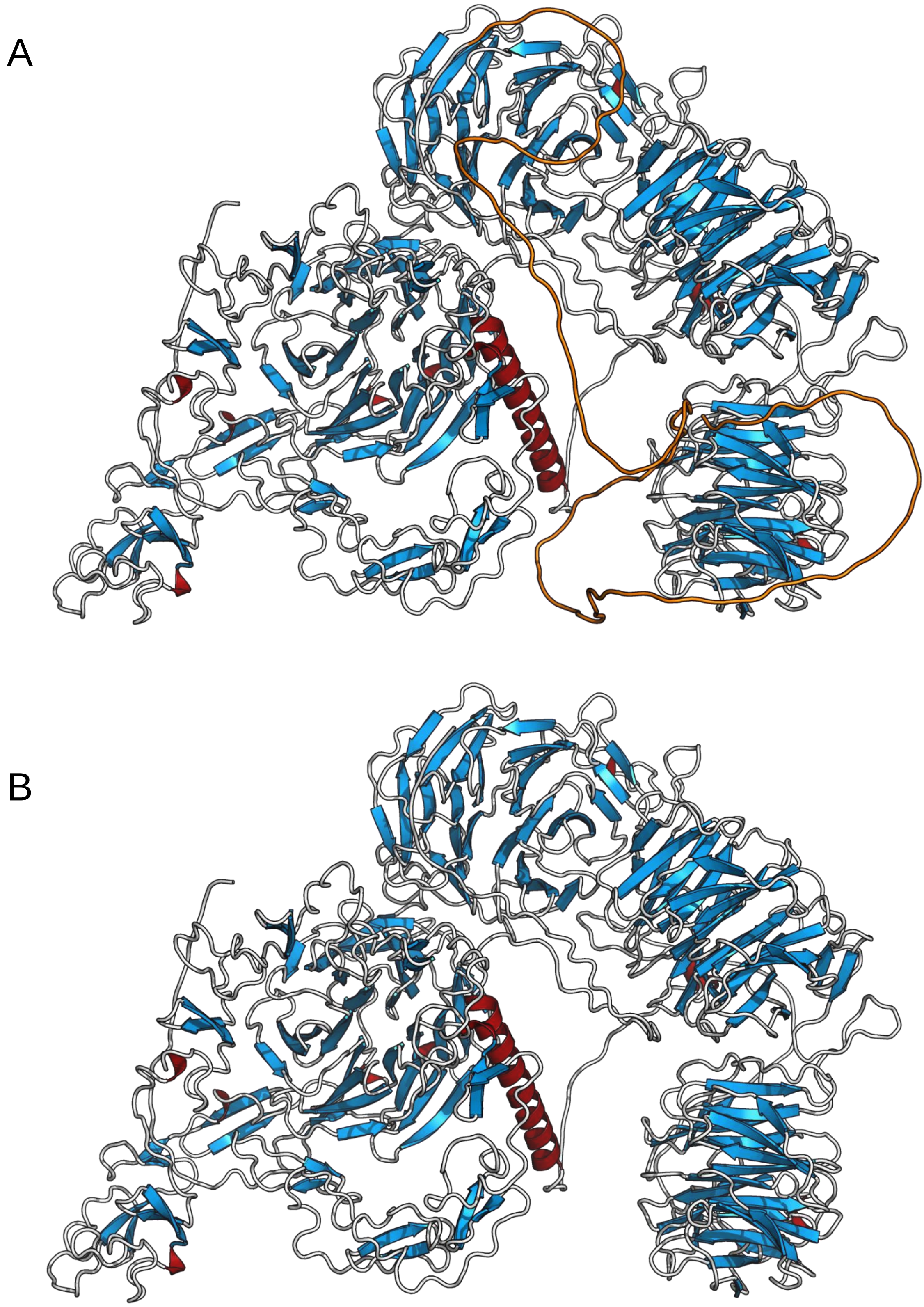

Fig. S9. Impact of exon 37b on LRP4. (A) 3D structure of LRP4 without exon 37B, the amino acids that are lost when exon 37B is included are colored in orange. **(B)** 3D structure of LRP4 with exon 37B.

Table S1. Differential expression analysis of early muscle maturation

Available for download at
https://journals.biologists.com/dmm/article-lookup/doi/10.1242/dmm.052098#supplementary-data

Table S2. Differential expression analysis of late muscle maturation

Available for download at
https://journals.biologists.com/dmm/article-lookup/doi/10.1242/dmm.052098#supplementary-data

Table S3. Enrichment analysis of upregulated genes during early muscle maturation

Available for download at
https://journals.biologists.com/dmm/article-lookup/doi/10.1242/dmm.052098#supplementary-data

Table S4. Enrichment analysis of downregulated genes during early muscle maturation

Available for download at
https://journals.biologists.com/dmm/article-lookup/doi/10.1242/dmm.052098#supplementary-data

Table S5. Enrichment analysis of upregulated genes during late muscle maturation

Available for download at
https://journals.biologists.com/dmm/article-lookup/doi/10.1242/dmm.052098#supplementary-data

Table S6. Enrichment analysis of downregulated genes during late muscle maturation

Available for download at
https://journals.biologists.com/dmm/article-lookup/doi/10.1242/dmm.052098#supplementary-data

Table S7. Enrichment analysis of alternatively spliced genes during early muscle maturation

Available for download at
https://journals.biologists.com/dmm/article-lookup/doi/10.1242/dmm.052098#supplementary-data

Table S8. Enrichment analysis of alternatively spliced genes during late muscle maturation

Available for download at
https://journals.biologists.com/dmm/article-lookup/doi/10.1242/dmm.052098#supplementary-data

Table S9. Evaluation of DE and AS in genes associated to the RNA splicing GO term

Available for download at
https://journals.biologists.com/dmm/article-lookup/doi/10.1242/dmm.052098#supplementary-data

Table S10. Enrichment analysis of DE-specific genes

Available for download at
https://journals.biologists.com/dmm/article-lookup/doi/10.1242/dmm.052098#supplementary-data

Table S11. Enrichment analysis of AS-specific genes

Available for download at
https://journals.biologists.com/dmm/article-lookup/doi/10.1242/dmm.052098#supplementary-data

Table S12. Enrichment analysis of genes affected by DE and AS

Available for download at
https://journals.biologists.com/dmm/article-lookup/doi/10.1242/dmm.052098#supplementary-data

Table S13. Number of AS events detected in myopathy-associated genes during early and late skeletal muscle maturation

Available for download at
https://journals.biologists.com/dmm/article-lookup/doi/10.1242/dmm.052098#supplementary-data

Table S14. RT-PCR primers

Gene	Primer sequence	Product size (bp)
Lrp4 37B	*F* 5′ CTCTTCCATCCCTCTGATAGCCTTTCACC 3′	473
	R 5′ CGCGTTGTTATGTGAGCACAAGGATCAG 3′	
LRP4 37B	F 5′ AGAAAGAG/CAGCTCTTTCACCCCTCTG 3′	299
	R 5′ CTCAGACTGCTCTTCCTGTAACAG 3′	
